# Study on the Potential Mechanism of Bisphenol A-induced Human Diabetic Nephropathy Based on Network Toxicology and Molecular Docking

**DOI:** 10.2174/0118715303467295260507193302

**Published:** 2026-05-21

**Authors:** Jiaxin Li, Xiuqin Ni, Qi Fan, Fuli Jian

**Affiliations:** 1 Jiangsu Food & Pharmaceutical Science College, School of Health Medicine, Huai’an 223003, China;; 2 Heilongjiang University of Chinese Medicine, Harbin 150000, China;; 3 The Thirteenth People’s Hospital of Chongqing, Chongqing 400053, China

**Keywords:** Bisphenol A, diabetic nephropathy, network toxicology, molecular docking, BPA-induced nephrotoxicity, therapeutic strategies

## Abstract

**Introduction:**

This study aims to systematically elucidate the potential molecular targets and core signaling pathways underlying bisphenol A (BPA)-induced diabetic nephropathy (DN) through the integration of network toxicology and molecular docking approaches.

**Methods:**

Using multiple public databases—including ADMETlab 2.0, ProTox-III, SwissTargetPrediction, GeneCards, OMIM, TTD, and the PDB—we systematically identified the potential therapeutic targets of BPA and the disease-associated targets of DN. Overlapping targets between the two sets were rigorously extracted using Venn diagram analysis. Subsequently, a BPA-DN protein-protein interaction network was constructed and visualized using Cytoscape. Functional enrichment analyses (GO and KEGG) were performed to clarify the biological processes and signaling pathways involved. Finally, molecular docking simulations between BPA and top-ranked hub targets were conducted using CB-Dock2 to evaluate binding feasibility and stability.

**Results:**

A total of 42 potential therapeutic targets of BPA in DN were identified through integrative bioinformatic analysis. Functional enrichment analysis indicated that immune-inflammatory response and programmed cell death were the predominant biological processes associated with BPA-induced toxicity. Molecular docking simulations demonstrated stable binding conformations between BPA and six core targets—ESR1, PTGS2, MMP2, BCL2, MMP9, and INS—with binding energies ranging from -6.1 to -8.6 kcal/mol, suggesting strong binding affinity. Pathway enrichment analysis further indicated that the MAPK, PI3K-Akt, and NF-κB signaling pathways act as central mediators in BPA-associated DN pathogenesis.

**Discussion:**

This study clarifies the molecular mechanism by which BPA may induce DN through multitarget and multipathway approaches. The findings not only provide a new theoretical framework for understanding the pathogenesis of BPA-induced nephrotoxicity but also demonstrate the effectiveness of network toxicology in identifying toxic pathways of environmental pollutants. Moreover, they offer a scientific basis for the prevention of BPA-related diabetic complications and the development of targeted therapeutic strategies.

**Conclusion:**

BPA may influence the secretion and sensitivity of INS by interfering with the ESR1-mediated hormone signaling pathway, affecting the expression of PTGS2, MMP2, and MMP9, modulating BCL2, and ultimately promoting the pathological progression of DN.

## 1 INTRODUCTION

Diabetes mellitus (DM) is a complex chronic disease caused by an absolute or relative deficiency of insulin, including type 1 diabetes mellitus (T1DM) and type 2 diabetes mellitus (T2DM). There are nearly 500 million diabetic patients worldwide, and this number is expected to increase by 25% and 51% by 2030 and 2045, respectively, posing a serious public health challenge [1]. In this context, DN is a particularly prominent complication and is the primary cause of chronic kidney disease (CKD) and end-stage renal disease (ESRD). About 30%~40% of diabetic patients will be affected [2]. Patients with DN frequently depend on dialysis or kidney transplantation to sustain life. This condition not only imposes a substantial economic and psychological burden on patients but also consumes considerable medical resources. Consequently, exploring effective strategies to improve the prognosis of DN is of great clinical significance [3].

With the increasing incidence of chronic diseases, environmental exposure to exogenous compounds such as BPA is also rising [4]. BPA serves as a fundamental raw material for the production of polycarbonate plastics and epoxy resins. It is widely detected in food packaging, medical devices, heat-sensitive paper, and even textiles, resulting in extensive population exposure [5]. It is well established that BPA exhibits endocrine - disrupting effects. Due to its physicochemical properties, it can cross physiological barriers, such as the blood - brain barrier and the placental barrier [6]. Among various organs, the kidney is one of the primary targets for BPA accumulation. Under long - term exposure, BPA concentration in the kidney increases progressively, indicating a cumulative effect. Human epidemiological studies have shown that elevated urinary BPA levels are associated with an increased risk of proteinuria [7]. In addition, animal studies have confirmed that a five - week intraperitoneal injection of BPA can induce podocyte injury and increase urinary albumin excretion in mice, further supporting its renal toxicity [8].

In recent years, increasing attention has been directed toward the adverse effects of BPA on kidney health. The mechanisms underlying renal injury have been partly clarified and involve disruption of mitochondrial homeostasis, induction of oxidative stress, and activation of inflammatory responses [9]. At the clinical level, serum BPA levels have been identified as a potential biomarker for predicting the prognosis of CKD in patients with T2DM [10]. However, the specific molecular mechanisms of BPA-induced DN, particularly the gene expression alterations it mediates and the characteristics of the immune response, remain to be fully elucidated. This issue requires further investigation. Such conditions provide an opportunity for systematic analysis of the pathogenic mechanisms of BPA in DN at the genomic level. Therefore, in high-risk populations such as individuals with diabetes and renal insufficiency, in - depth investigation of the role of BPA in DN pathogenesis has both significant theoretical value and clear clinical implications.

Network toxicology provides a systematic research approach for analyzing the complex toxicity mechanisms of chemical exposure through integration of multi-omics data and computational models [11]. This approach enables the construction of interaction networks among compounds, toxicological endpoints, and biological targets, thereby transforming complex multi-component and multi-target relationships into intuitive visual models and identifying key toxic pathways. Based on target recognition, molecular docking, as an important computational technique, can predict the interaction mode and binding affinity between ligands and proteins [12], and it is of great value for elucidating the molecular mechanisms underlying the toxicity of environmental pollutants such as BPA. Although its application still faces challenges—particularly in balancing prediction accuracy and structural interpretability—the integration of machine learning algorithms has significantly improved predictive performance. Based on the above-established methodological framework, molecular docking was employed in this study to analyze the interaction between BPA and kidney-related proteins. Although these computational tools have been widely used in toxicology research, the integration of network toxicology and molecular docking to explore the mechanism of BPA-induced DN remains limited—this constitutes the key scientific problem addressed in this study.

In this study, network toxicology and molecular docking techniques were employed to systematically elucidate the toxic molecular mechanisms underlying BPA-induced DN. By integrating multi-omics data with computational models, key molecular targets and signaling pathways involved in the pathogenic role of BPA in DN were identified and experimentally validated. This study not only provides new evidence for understanding the health risks posed by environmental toxicants but also offers a theoretical basis for identifying therapeutic targets in related diseases.

## 2 MATERIALS AND METHODS

### 2.1 The Network Toxicology Analysis of BPA

To systematically evaluate the toxicological characteristics of BPA, computational toxicology methods were employed to integrate two established prediction platforms. ADMETlab 2.0 was used to comprehensively predict pharmacokinetic properties—including absorption, distribution, metabolism, and excretion (ADME)—while ProTox-III was applied for an in-depth assessment of multi-endpoint toxicity. The molecular structure of BPA was represented by its standardized SMILES (Simplified Molecular Input Line Entry System) identifier, obtained from the PubChem database (https://pubchem.ncbi.nlm.nih.gov), ensuring authoritative and consistent chemical representation. This SMILES string was imported as a unified input into both platforms. All calculations were conducted using default parameters, with the species specified as *Homo sapiens* to ensure physiological relevance and cross-study comparability of the predictions. Finally, toxicological prediction data from ProTox-3.0 (https://tox.charite.de/) and ADMETlab 2.0 (https://admetmesh.scbdd.com/) were combined for systematic integration and cross-validation, providing a reliable data foundation for the subsequent construction of the compound-target-toxicity network.

### 2.2 Identification of BPA Targets

To comprehensively identify the potential molecular targets of BPA, two complementary strategies were adopted. First, experimentally validated protein targets were retrieved from the ChEMBL database (https://www.ebi.ac.uk/chembl/), and their UniProt identifiers were converted to standard gene symbols using the official UniProt ID mapping service. Second, the SMILES structure of BPA was submitted to the SwissTargetPrediction server (http://www.swisstargetprediction.ch/), enabling structure-based reverse docking prediction. At this stage, all predicted targets with probability scores > 0 were retained in the preliminary candidate list. Finally, targets from both sources were integrated, and duplicate entries—defined as identical gene symbols—were removed to generate a non-redundant target set. In cases where conflicting evidence existed for the same target across databases, experimentally validated entries from ChEMBL were prioritized. All analyses were restricted to human proteins to ensure biological relevance. This integrated strategy yielded a high-confidence set of potential molecular targets for subsequent mechanistic analysis.

### 2.3 Construction of DN Target Library

To construct a disease target library related to DN, data from three authoritative databases were systematically integrated: GeneCards (https://www.genecards.org/), OMIM (https://omim.org/), and TTD (http://db.idrblab.net/ttd/). The specific procedure was as follows: First, the GeneCards database was searched using the keyword “diabetic nephropathy”. To ensure both analytical feasibility and biological relevance, a GeneCards relevance score > 10 was adopted as the screening threshold—this cutoff was informed by previous similar studies and supported by preliminary sensitivity analysis. Next, the OMIM and TTD databases were queried using the same keyword to retrieve additional targets supported by experimental or clinical literature evidence and/or annotated as drug targets. Finally, the target lists from all three databases were merged, and duplicates were rigorously removed to obtain a comprehensive, non - redundant, DN-specific target set. This systematic and carefully validated screening strategy prioritizes high-confidence targets and establishes a solid basis for subsequent network pharmacological analysis.

### 2.4 Intersection Analysis of Compounds and Disease Targets

To identify the direct molecular association between BPA targets and DN, a systematic intersection analysis of the two gene sets was performed using the ggvenn R software package. The pre-processed BPA-specific target gene set and the DN-related gene set were used as input files, and common target genes were identified through Venn diagram analysis. Based on the resulting intersection gene set, the following core data were generated: (1) a curated list of common target genes; (2) network files describing interactions between BPA - associated and DN-associated genes; (3) annotation files containing node type and attribute information—providing a basis for subsequent network visualization and topological analysis.

### 2.5 Construction of Complex Disease Regulatory Network

To systematically characterize the complex interactions between BPA-responsive genes and DN-related targets, Cytoscape was used to construct a comprehensive regulatory network. This network integrates gene targets significantly regulated by BPA exposure with known DN-related genes. Based on this network, its topological properties were analyzed to identify hub nodes that play central regulatory roles.

### 2.6 GO and KEGG Pathway Enrichment Analysis

To elucidate the biological functions of the common targets of BPA and DN, Gene Ontology (GO) enrichment analysis and Kyoto Encyclopedia of Genes and Genomes (KEGG) pathway analysis were performed using the R package clusterProfiler on the 42 overlapping genes. The significance threshold was set at p < 0.05, and a false discovery rate (FDR)-corrected p < 0.05. Results were visualized across the three GO categories: biological processes (BP), cellular components (CC), and molecular functions (MF).

### 2.7 PPI Network Construction

To analyze the interaction between BPA and its common targets at the protein level, a protein-protein interaction (PPI) network was constructed based on the STRING database. First, a list of 42 overlapping genes identified by Venn analysis was uploaded to the STRING platform (https://string-db.org/), with the organism set to “Homo sapiens” to obtain high-confidence interaction data. Next, the resulting TSV-formatted network file was imported into Cytoscape for visualization and topological analysis. Finally, the Cytoscape NetworkAnalyzer plugin was used to calculate topological properties for each node; nodes were visualized according to the “degree” centrality metric—node size and color intensity were scaled proportionally to degree values, enabling intuitive identification of hub proteins with the highest connectivity, which are likely to occupy central regulatory positions in the network.

### 2.8 Molecular Docking Verificatio**n**

Six target proteins with the highest degree values from the PPI network were selected for molecular docking. First, the three-dimensional crystal structures of these target proteins were downloaded from the PDB (https://www.rcsb.org/) using “Homo sapiens” as the species filter. Then, the receptor proteins were preprocessed (*e.g.*, removal of water molecules and heteroatoms, protonation, and energy minimization) before docking. Subsequently, the pretreated receptor proteins and the BPA ligand were submitted to CB-Dock2 (https://cadd.labshare.cn/cb-dock2/index.php), an online platform for blind docking analysis. After docking, the top five binding poses (*i.e.*, conformations) with the lowest predicted binding free energy were extracted for each protein-BPA complex. Lower binding free energy indicates a more thermodynamically favorable and stable protein-ligand interaction.

## 3 RESULTS

### 3.1 Analysis of BPA Toxicity Results

To elucidate the molecular mechanism of BPA (CAS:80-05-7) -induced DN, a comprehensive computational approach was employed to identify its potential molecular targets. This approach integrates public database mining and structure-based toxicity prediction strategies. First, standardized chemical information of the compound was obtained from the PubChem database, including its molecular formula (C_15_H_16_O_2_), standard SMILES representation of CC(C)(C1 =CC=C(C=C1)O)C2 =CC=C(C=C2)O, and its 2D and 3D chemical structures, providing an accurate molecular characterization basis for subsequent analysis. Furthermore, the ProTox database was used to predict systemic toxicity. BPA treatment can induce a decrease in mitochondrial membrane potential, suggesting interference with mitochondrial function and cellular energy metabolism. Further molecular docking and enzyme activity prediction analysis showed that acetaldehyde exhibited a significant activation effect on cytochrome CYP2C9, indicating possible interference with enzyme-related metabolic pathways. The predictive analysis of toxicological properties using the ADMETlab 2.0 platform showed that BPA has potential eye irritation and skin toxicity, indicating that it may cause direct damage to local tissues. Overall, the integrated prediction results indicate that BPA may exert toxic effects by altering mitochondrial membrane potential, interfering with key metabolic enzymes, and inducing local tissue toxicity. Existing literature supports the established association between BPA and DN [13]. Based on the above findings, the specific molecular mechanisms of BPA in DN pathogenesis will be the focus of subsequent sections, where a systematic and in-depth discussion will be presented.

### 3.2 Target Analysis of BPA

**Fig. (1):**
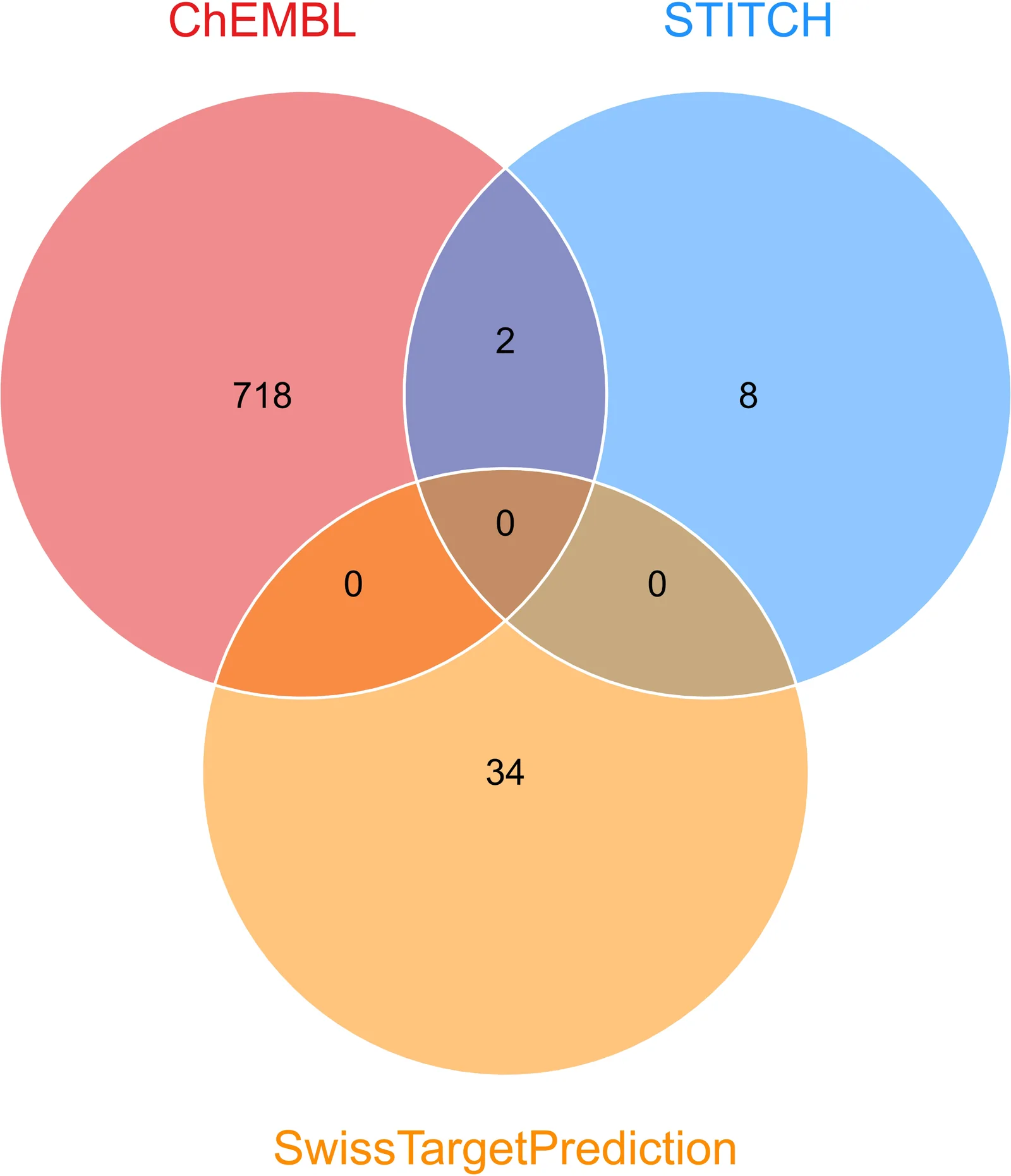
Venn diagram of the BPA target gene. Integrated from ChEMBL, STITCH, and the SwissTargetPrediction database.

This study systematically constructed the potential target set of BPA. Experimentally validated BPA targets were first extracted from the ChEMBL database, followed by target prediction and interaction network construction using the STITCH database. Concurrently, additional predicted targets were identified through the SwissTargetPrediction database. To integrate these datasets, Venn analysis was performed across all target sets, and a high-confidence BPA target set was ultimately defined by merging the results and removing duplicates (Fig. **1**).

### 3.3 Identification of DN-related Genes

**Fig. (2):**
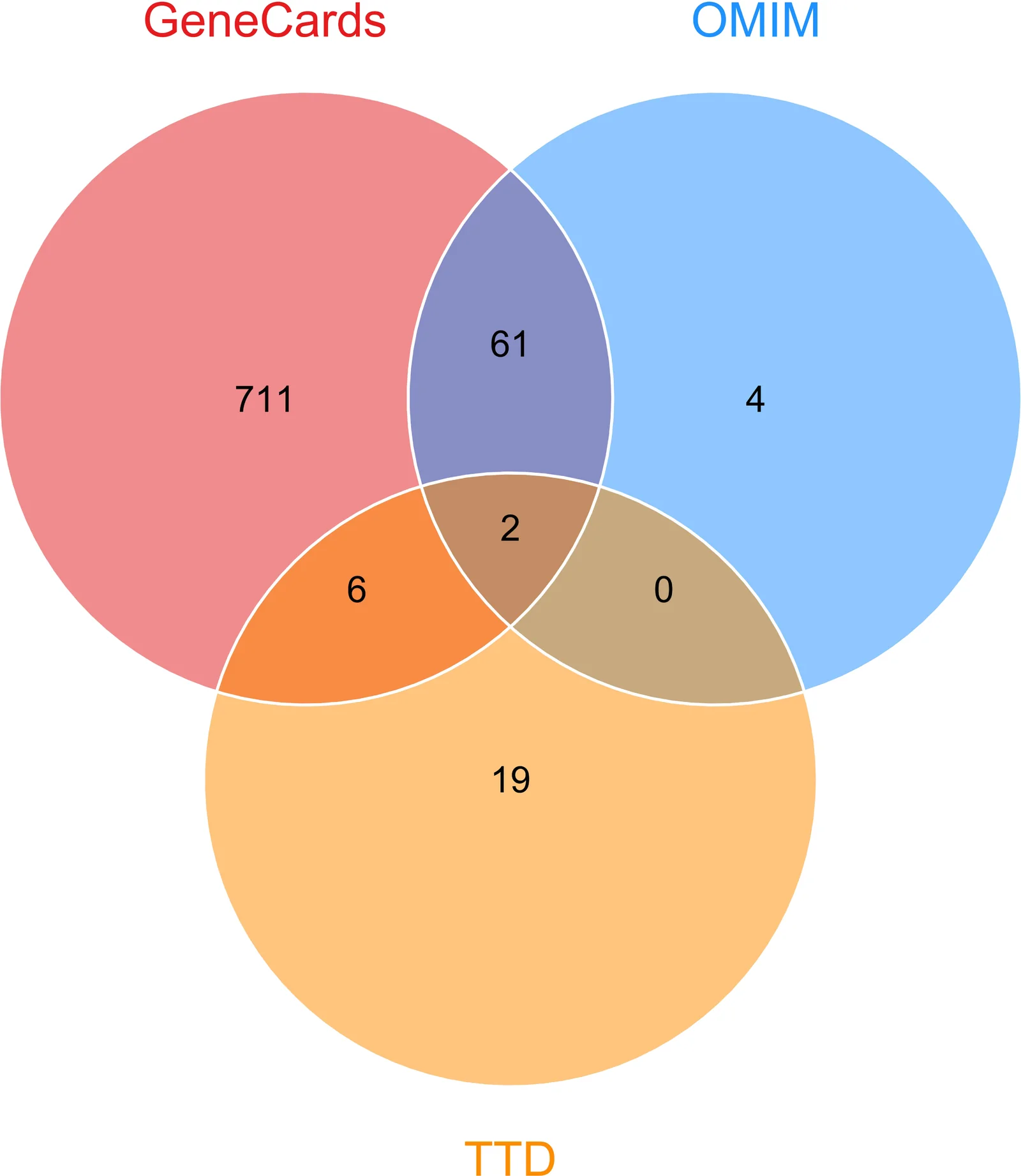
Venn diagram of DN target gene. Integrated from GeneCards, OMIM, and TTD databases.

To systematically identify DN-related genes, information from three authoritative databases—GeneCards, OMIM, and TTD—was integrated. First, using a preset correlation score threshold (>10), 780 high-correlation genes were screened from the GeneCards database. Next, 67 and 27 DN-related genes supported by literature or experimental evidence were retrieved from OMIM and TTD, respectively. By integrating these gene sets and removing duplicates, a non-redundant DN-related gene set comprising 803 unique genes was obtained (Fig. **2**), which was subsequently used for Venn diagram analysis and network construction.

### 3.4 Potential Core Targets of BPA-induced DN

**Fig. (3):**
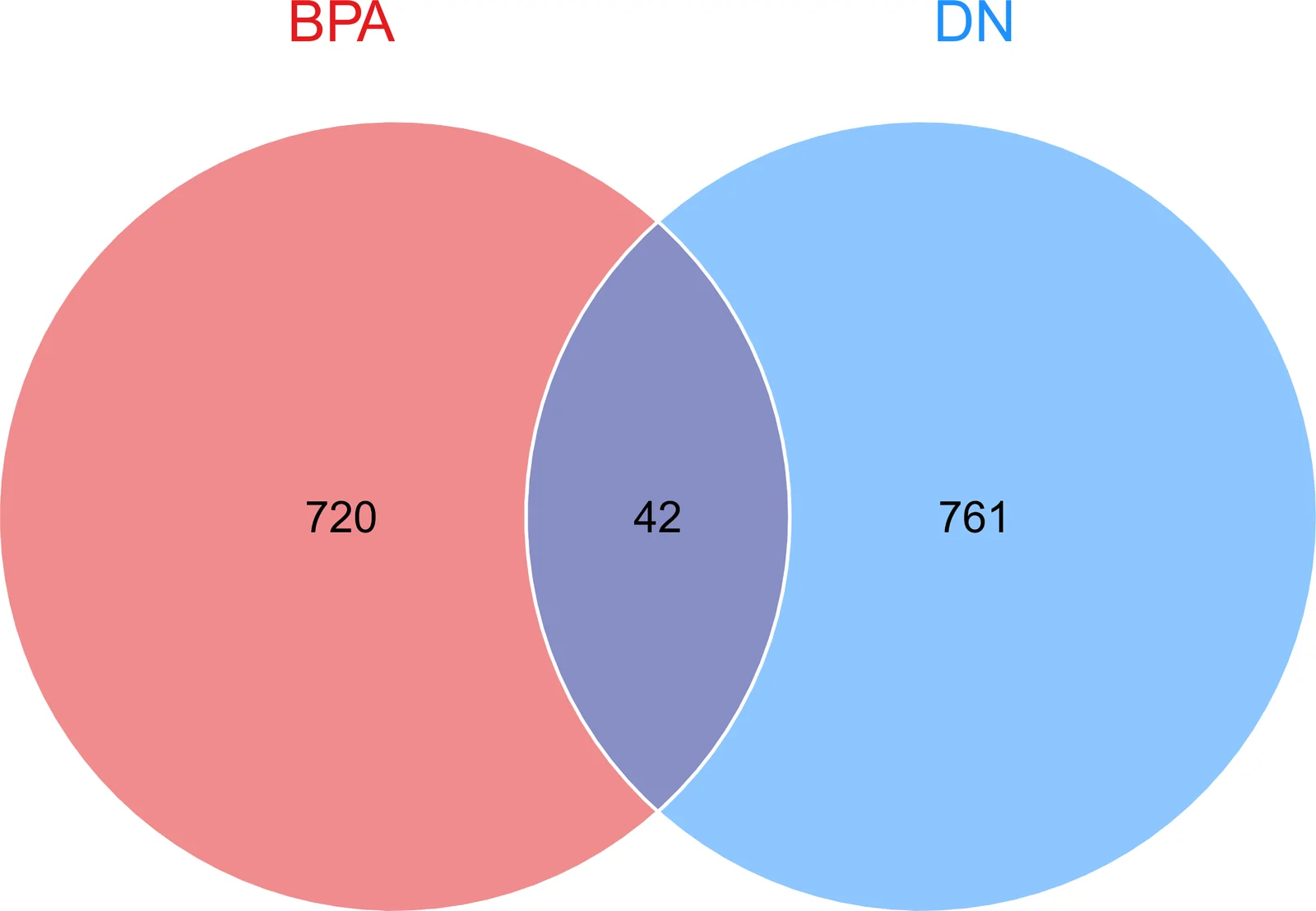
Venn diagram of compound target genes and disease-related genes.

To reveal the potential molecular mechanisms underlying BPA-induced DN, 803 DN-related genes and 762 BPA-associated targets were systematically integrated and compared. Venn diagram analysis identified 42 overlapping genes between the two datasets (Fig. **3**). These intersecting genes were regarded as core candidate targets through which BPA may influence the pathogenesis of DN.

### 3.5 BPA-DN Interaction Regulatory Network

**Fig. (4):**
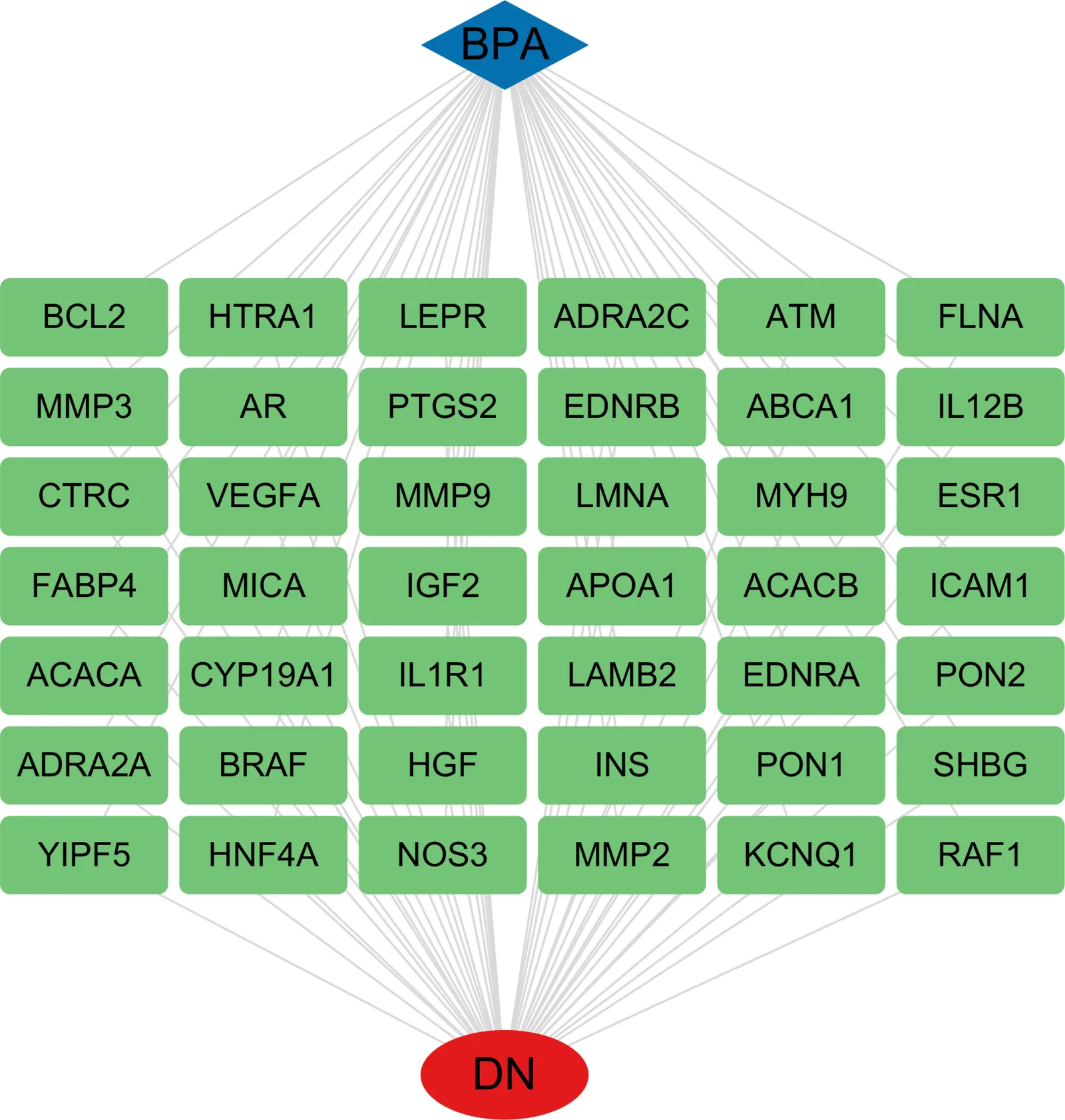
Molecular interaction network of BPA-induced DN. This network diagram illustrates the “compound-target-disease” interaction relationships. Among the nodes, 42 overlapping targets shared between BPA and DN are highlighted with green rectangles; BPA is represented by blue diamonds; and the DN phenotype is denoted by red ellipses. The integrated network reveals potential molecular pathways through which BPA may contribute to DN pathogenesis by modulating these core targets.

To systematically clarify the molecular associations between BPA and DN, a comprehensive regulatory network comprising BPA, its target genes, and DN-related genes was constructed based on the common targets identified above. The network was visualized using the Cytoscape platform, with differentiated visual attributes (*e.g.,* color coding and node shape) used to distinguish different types of network components. An optimized layout algorithm was applied to clearly present the topological structure (Fig. **4**). This systems biology approach provides an intuitive representation of the complex interactions between BPA exposure and DN pathogenesis.

### 3.6 GO Enrichment Analysis Results

**Fig. (5):**
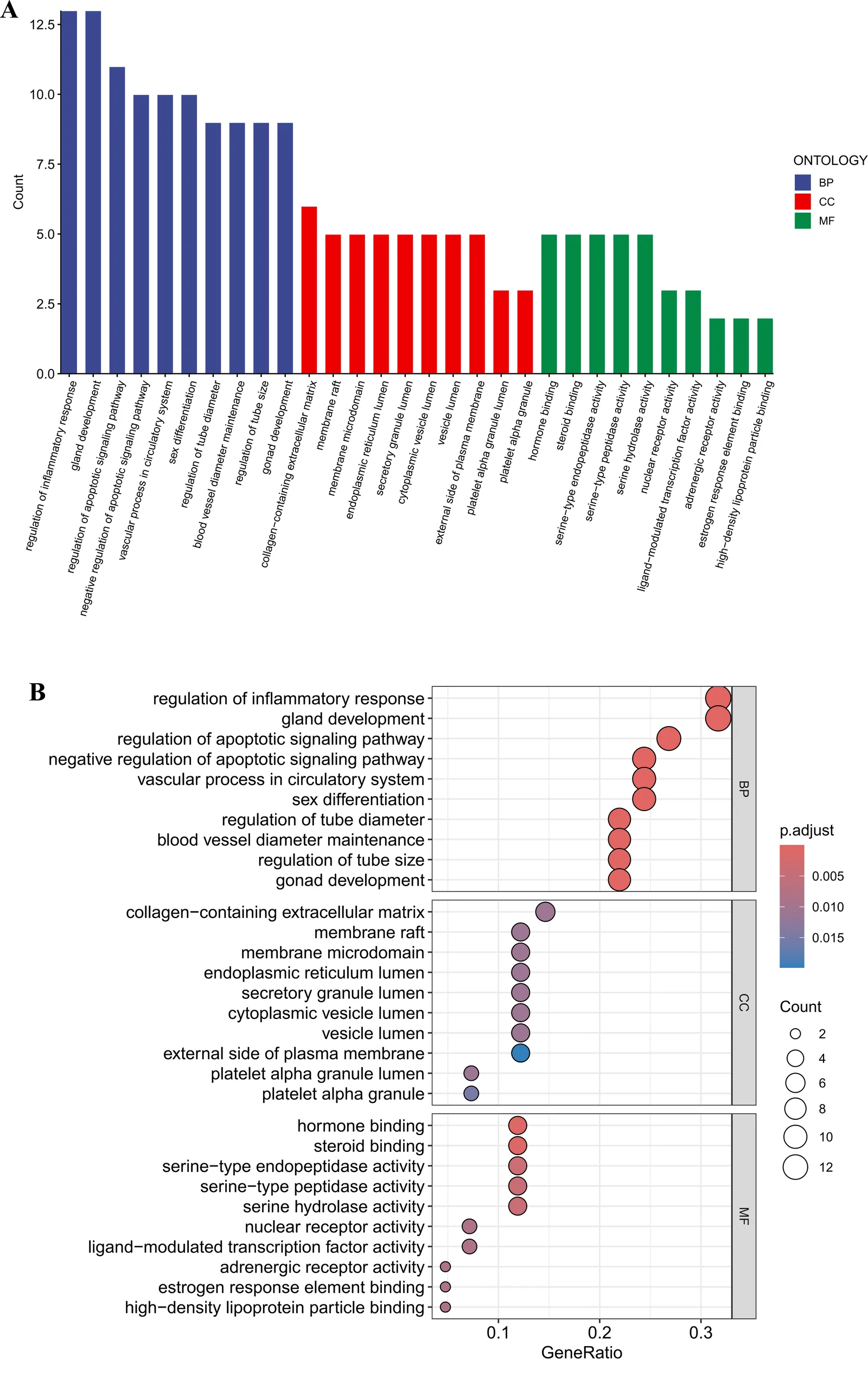
Functional enrichment analysis of BPA-DN overlapping genes. (**A**) GO enrichment analysis results bubble diagram. The GO entries in the top 10 of the significance ranking were displayed, the bubble color depth represented-log10 (p value), and the bubble size represented the number of enriched genes. (**B**) Enrichment analysis bar chart.

To systematically interpret the biological functions of the common targets shared by BPA and DN, GO enrichment analysis was performed on the 42 overlapping genes using the clusterProfiler R package. The results were ranked by adjusted p value (Fig. **5A**), revealing 831 BP, 22 CC, and 55 MF terms that were statistically significantly enriched in relation to BPA-induced DN. (Fig. **5B**) presents the top 10 most significant terms in each category.

At the BP level, the core enriched terms mainly involved regulation of inflammatory response, regulation of apoptotic signaling pathway, and vascular processes in the circulatory system—indicating that BPA may contribute to DN progression by modulating immune-inflammatory responses, programmed cell death, and vascular function. CC analysis showed significant enrichment of target genes in collagen-containing extracellular matrix, endoplasmic reticulum lumen, and related structures, reflecting their roles in maintaining extracellular microenvironment stability and endoplasmic reticulum-associated protein processing. In the MF category, terms such as hormone binding and steroid binding were significantly enriched, suggesting that BPA—as an environmental endocrine disruptor—may exert biological effects by mimicking or interfering with endogenous hormone functions.

### 3.7 KEGG Pathway Enrichment Analysis Results

**Fig. (6):**
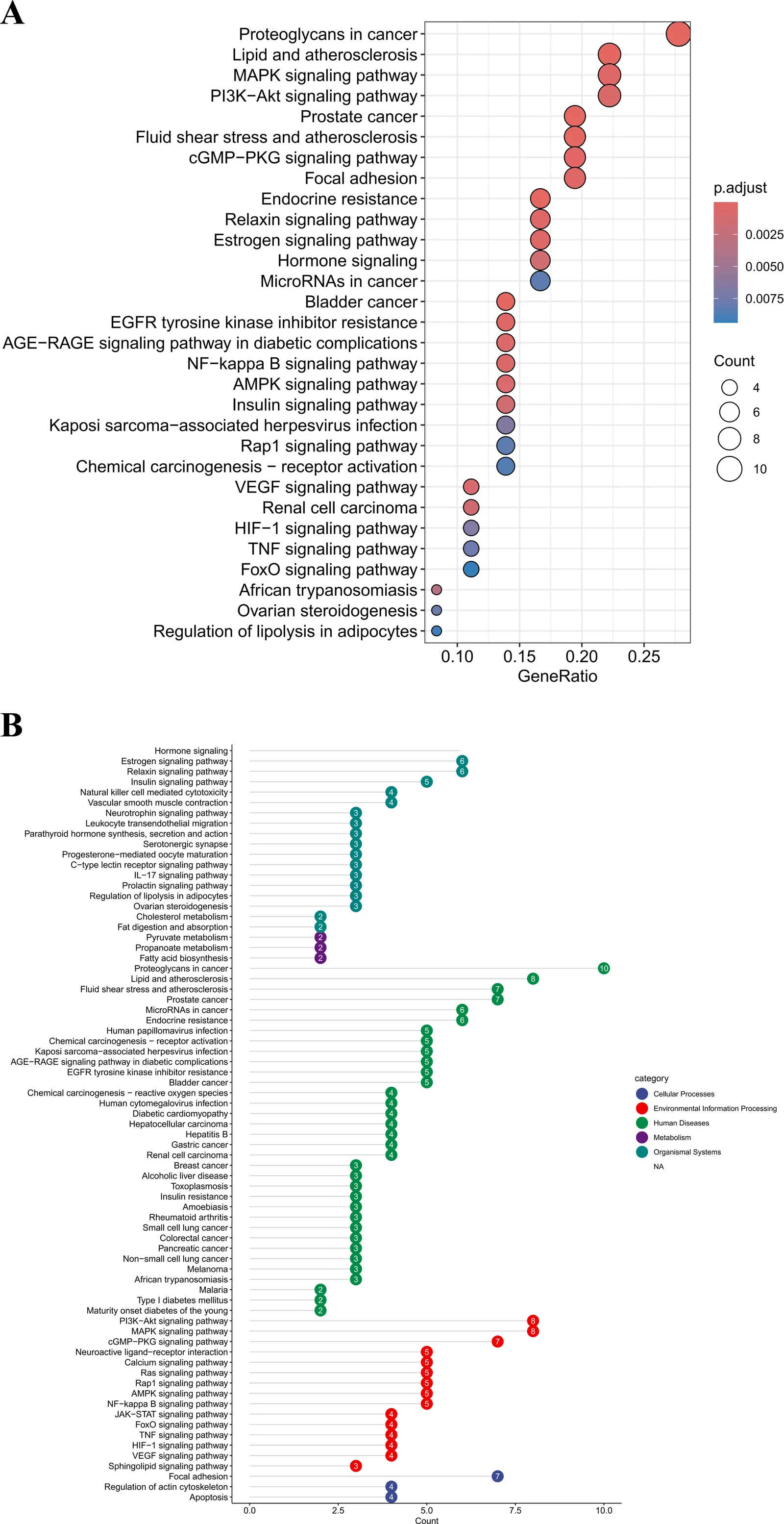
KEGG pathway enrichment analysis of BPA-DN overlapping genes. (**A**) KEGG pathway enrichment significance ranking diagram based on adjusted p value. (**B**) KEGG pathway enrichment bar chart. The color of the circle superimposed on the bar represents the first-level functional category of the pathway, the size of the circle represents the number of genes enriched in the pathway, and the x-axis label is the specific pathway name.

To systematically elucidate the signal transduction and metabolic pathways associated with the common targets of BPA and DN, KEGG pathway enrichment analysis was performed. A corrected p < 0.05 after FDR correction was used as the significance threshold, and several signaling pathways closely related to the pathogenesis of BPA-induced DN were identified. As shown in Fig. (**6A**), the overlapping genes were significantly enriched in key pathways—including the MAPK signaling pathway, the PI3K-Akt signaling pathway, the NF-κB signaling pathway, the AMPK signaling pathway, and the insulin signaling pathway (Fig. **6A**).

These genes are mainly distributed across six KEGG functional categories: Cellular Processes, Environmental Information Processing, Human Diseases, Metabolism, Organismal Systems, and “Unclassified” (though the latter is not explicitly labeled in the original text; consistent with standard KEGG annotation practice, “Unclassified” is often included—however, since it is absent from the original list and no evidence supports its presence here, it should be omitted unless verified). Notably, these categories include multiple disease-related pathways directly associated with metabolic disorders, endocrine dysregulation, and inflammatory responses (Fig. **6B**). Collectively, these findings indicate that BPA may contribute to the onset and progression of DN by disrupting the coordinated regulation of multiple interconnected biological systems and signaling pathways.

### 3.8 Screening of Key Core Genes of BPA-mediated DN

**Fig. (7):**
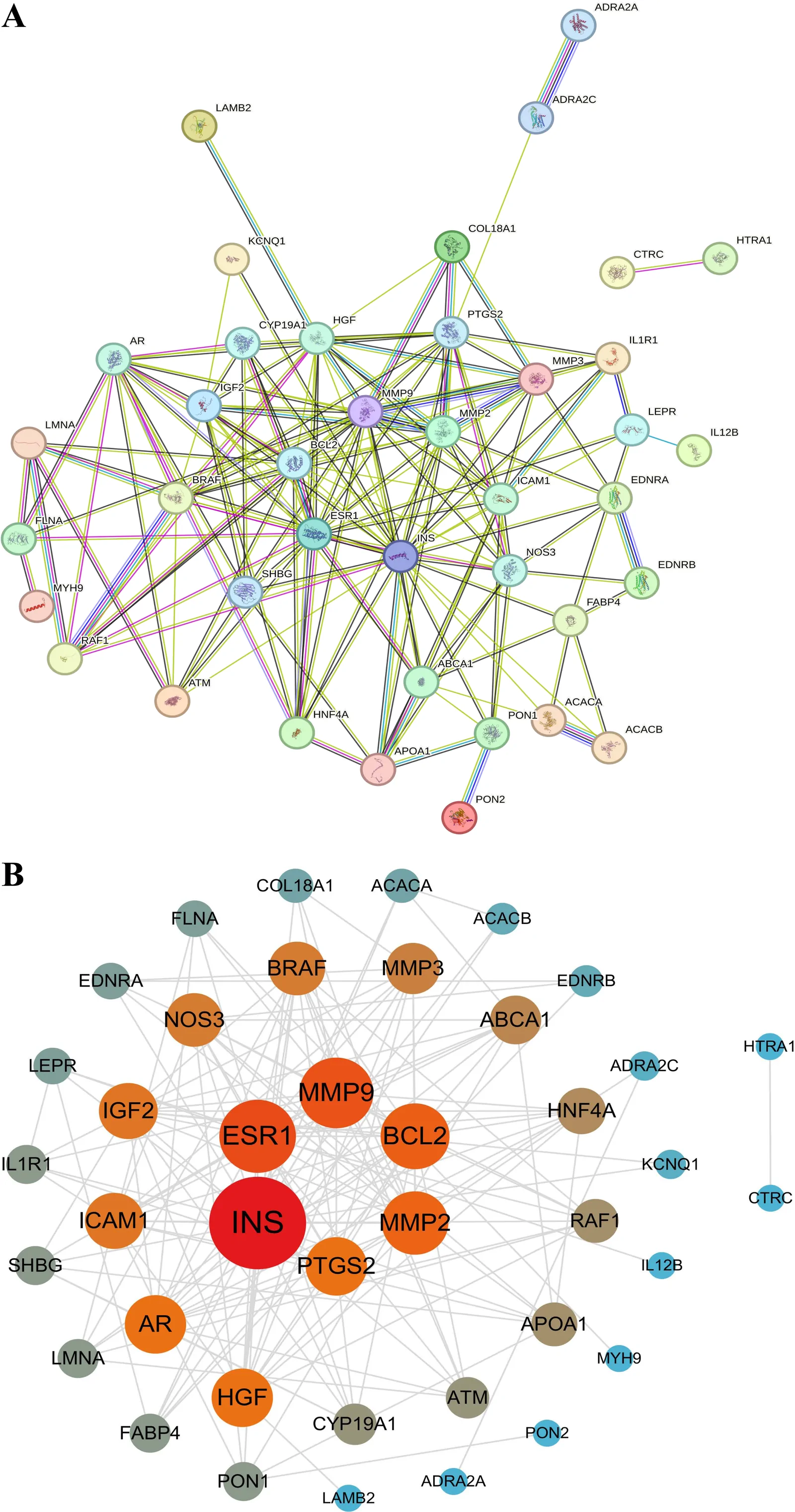
shows the gene interaction network of BPA-induced DN. (**A**) PPI network diagram, in which nodes with higher connectivity suggest that they may represent core targets in the network; (**B**) Core gene network. The node size in the graph is proportional to the node degree value. The larger the node, the broader the connectivity in the network, indicating a potential central role in functional regulation.

To identify the most influential core genes from the PPI network, the STRING database was first used to construct an interaction network of overlapping targets (Fig. **7A**), which was then imported into Cytoscape for topological analysis. By calculating the degree centrality of nodes (*i.e.*, the number of connections per node), key genes with the highest connectivity and potential central roles in the network were identified. As shown in Fig. (**7B**), the final set of core genes includes INS, ESR1, MMP9, BCL2, MMP2, and PTGS2. These genes exhibited very high connectivity in the network, suggesting that they may occupy central regulatory positions in the molecular network underlying BPA - induced DN.

### 3.9 Molecular Docking Results And Biological Interpretation

**Fig. (8):**
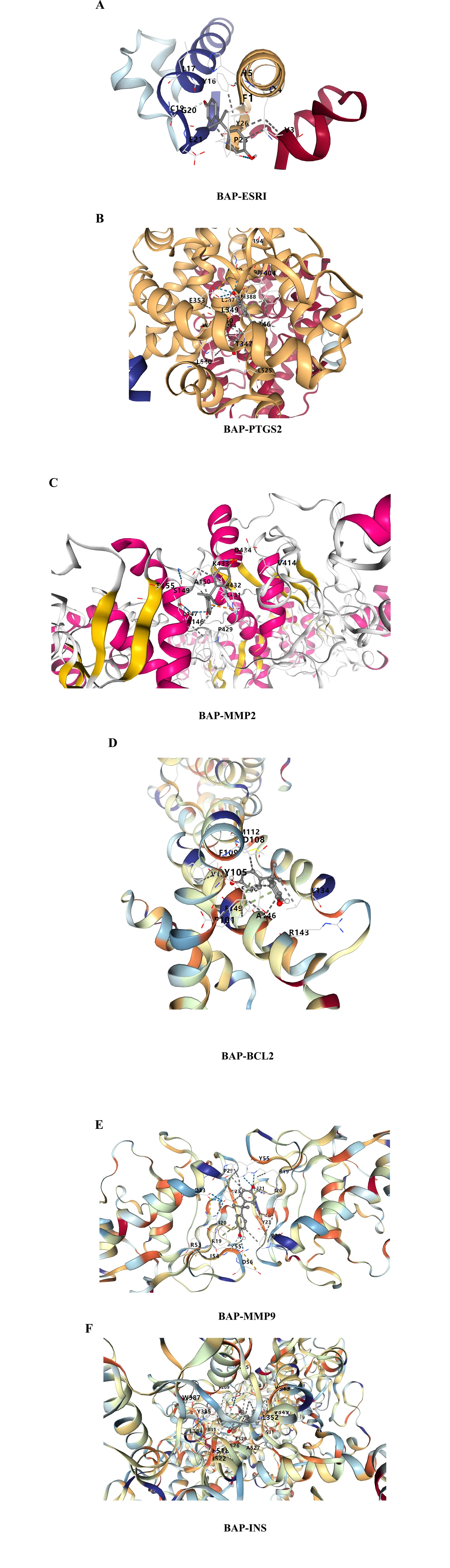
Molecular docking analysis of BPA and DN-related key proteins. (**A**) BPA and ESR1 (binding energy:-8.6 kcal/mol). (**B**) The complex of BPA and PTGS2 (- 7.7 kcal/mol). (**C**) The complex of BPA and MMP2 (-7.6 kcal/mol). (**D**) The complex of BPA and BCL2 (- 7.1 kcal/mol). (**E**) The complex of BPA and MMP9 (-7.0 kcal/mol). (**F**) The complex of BPA and INS (-6.1 kcal/mol).

Molecular docking results showed that BPA can form stable complexes with six core targets associated with DN (Fig. **8A**-**F**). As shown in Table **1**, all binding affinities were lower than -5 kcal/mol, indicating that the binding process was spontaneous. Among them, BPA exhibited the strongest binding affinity with ESR1 (-8.6 kcal/mol). In addition, BPA formed a stable interaction with the catalytic domain of MMP9 (-7.0 kcal/mol), which may interfere with extracellular matrix remodeling. Strong binding to INS and PTGS2 (-6.1 and -7.7 kcal/mol, respectively) was also observed, providing a plausible structural basis for BPA-mediated interference with insulin signaling and inflammatory signal transduction. The binding affinities with BCL2 and MMP2 were -7.1 and -7.6 kcal/mol, respectively. Although these results are based on computational predictions and require experimental validation, they are consistent with the established mechanisms and clinical observations of BPA as an endocrine disruptor. All identified binding sites are located in functionally critical regions of their respective target proteins, and the protein structures used were derived from the PDB entries listed in Table **1**.

## 4 DISCUSSION

In this study, network toxicology, molecular docking, and systematic literature analysis were integrated to explore the potential molecular mechanisms of DN induced by BPA. As a typical environmental endocrine disruptor, BPA is widely used in the production of polycarbonate plastics and epoxy resins, and the population is broadly exposed through daily consumer products such as food packaging. Epidemiological evidence indicates that BPA exposure is strongly associated with metabolic disorders, including obesity, diabetes, and cardiovascular disease [14]. Although regulatory restrictions on BPA have gradually been implemented and its structural analogues, BPS and BPF, have been promoted as alternatives, existing studies indicate that these substitutes still exhibit endocrine - disrupting activity [15]. This study focused on BPA, aiming to clarify the molecular mechanism of DN induced by single exposure and to provide a theoretical basis for subsequent studies on the joint toxicity of compound pollutants.

Through systematic bioinformatics analysis, 42 common targets shared by BPA and DN were identified. Functional enrichment analysis showed that these targets were significantly enriched in key biological processes, including immune-inflammatory response, regulation of vascular function, and endoplasmic reticulum-related protein processing. KEGG pathway analysis further indicated that BPA may participate in the pathological process of DN by interfering with the MAPK, PI3K - Akt, NF - κB, AMPK, and insulin signaling pathways. These pathways have been recognized as core regulatory mechanisms in diabetic complications. Notably, the enrichment of the AMPK and insulin signaling pathways suggests that BPA may contribute to DN development by affecting energy metabolism and insulin sensitivity.

Based on subsequent network analysis, six core molecular targets (ESR1, PTGS2, MMP2, BCL2, MMP9, and INS) were screened. Molecular docking results demonstrated that BPA exhibited favorable binding affinity with these targets (ranging from -6.1 to -8.6 kcal/mol), indicating that their interactions may play important roles in the pathogenesis of DN.

Traditional toxicology research relies on animal experiments and epidemiological investigations. Although it provides an important foundation for chemical safety assessment [16], it has limitations, including high cost, ethical concerns, and limited mechanistic interpretation. With the rapid emergence of new chemical substances, traditional assessment methods are facing increasing challenges. By identifying molecular initiating events and key nodes within the adverse outcome pathway, network toxicology provides a novel paradigm for a more detailed understanding of toxicity mechanisms [17] and supports the construction of adverse outcome pathways (AOP), which is of considerable importance for regulatory toxicology [18, 19]. This study reflects this methodological advantage.

Estrogen receptor 1 (ESR1), an estrogen receptor, regulates multiple biomarkers. A substantial body of research has shown that ESR1 is closely associated with the onset and progression of cardiovascular diseases and retinal complications induced by diabetes, particularly in female patients with DM [20]. The ESR1 protein can inhibit the transcriptional activity of the interleukin-6 (IL-6) promoter mediated by NF-κB, thereby modulating inflammation, immunity, and stress responses [21]. However, studies on T2DM and ESRD among African Americans (AA) remain limited. Gallagher *et al*. demonstrated that variation in the ESR1 gene was significantly associated with T2DM-ESRD in AA [22]. Seventeen single nucleotide polymorphisms (SNPs) of ESR1 were genotyped within a 41 - kb region spanning from intron 1 to intron 2. One SNP, rs1033182, and a haplotype composed of six independent SNPs with high linkage disequilibrium (LD) showed a positive correlation with T2DM - ESRD. In this study, the high-affinity binding of BPA to ESR1 (with a binding energy of -8.6 kcal/mol) suggests that BPA may influence DN progression by interfering with the estrogen signaling pathway.

PTGS2, also known as cyclooxygenase-2 (COX-2), is an enzyme expressed in multiple regions of the mammalian kidney, including dense plaques, medullary interstitial cells, arteriolar endothelial cells, and glomerular podocytes [23]. PTGS2 promotes prostaglandin production through paracrine signaling to reduce the contraction of glomerular arterioles associated with filtration. COX - 2 inhibitors may impair this protective mechanism for maintaining glomerular perfusion, resulting in sustained contraction of small glomerular arteries and reduced renal perfusion. This contributes to understanding the relationship between PTGS2 and renal function. On the one hand, overexpression of PTGS2 may intensify the inflammatory response and cause renal damage; on the other hand, excessive inhibition of PTGS2 may increase the risk of hyperkalemia and renal failure. Zhou *et al*. reported that RAAS inhibitors, which regulate PTGS2 expression in renal tissues of DN, may be associated with acute kidney injury, although the exact mechanism remains unclear. The only consistent conclusion is that PTGS2 may increase the risk of acute renal failure when RAAS inhibitors act under DN conditions [24]. The binding of BPA to PTGS2 with an affinity of -7.7 kcal/mol indicates that it may be involved in DN pathogenesis by modulating inflammatory responses.

MMP2 and MMP9, as members of the matrix metalloproteinase family, maintain the balance between synthesis and degradation of the extracellular matrix, thereby preserving the structural and functional integrity of the glomeruli [25]. Studies have shown that MMP9 levels are significantly elevated in the urine, serum, and kidney tissues of patients with DN [25]. Overexpression of MMP9 can induce podocyte dedifferentiation and disrupt podocyte integrity [26]. At the same time, urinary MMP9 levels increase earlier than microalbuminuria, and serum MMP9 levels are negatively correlated with the estimated glomerular filtration rate (eGFR) [25]. In addition, knockout of the MMP9 gene significantly alleviated proteinuria and prevented the above structural changes in kidney disease [26]. Therefore, MMP9 serves as a reliable biomarker for early diagnosis of DN. Chen *et al*. also reported that MMP9 expression was significantly elevated in the high glucose (HG) group [27]. It was observed that MMP9 expression and activity increased in the early stage of DN and decreased in the late stage [28]. The binding of BPA to MMP2 (-7.6 kcal/mol) and MMP9 (-7.0 kcal/mol) suggests that BPA may participate in DN progression by interfering with extracellular matrix remodeling.

BCL2 is a key regulator of endogenous apoptosis and controls cell death by maintaining mitochondrial outer membrane permeability [29]. Under normal conditions, autophagy and apoptosis maintain a dynamic balance. A reduction in protective autophagy and an increase in podocyte apoptosis are major contributors to renal injury in DN [30, 31]. BCL2 plays an important role in acute kidney injury induced by ischemia and nephrotoxic drugs [32]. In patients with diabetic chronic kidney disease, current findings indicate a negative correlation between BCL2, creatinine, and glycosylated hemoglobin percentage (HbA1c). The increase in apoptosis may be related to elevated levels of BCL2 inhibitors/killers and reduced expression of BCL2. Notably, the inhibitory effect of oral inhibitors on BCL2 inhibitors can suppress the development of tubulointerstitial inflammation in mice [33]. The results of Mahmoud *et al*. support the concept that reduced BCL2 levels are involved in the pathogenic process of renal injury mediated by diabetic inflammatory mechanisms [34]. The binding of BPA to BCL2 with an affinity of -7.1 kcal/mol suggests that it may participate in the pathological process of DN by regulating apoptosis.

INS-encoded insulin serves as a central hormone maintaining blood-glucose homeostasis and energy metabolism and exhibits vascular - protective effects. Hyperglycemia suppresses the anti - atherosclerotic effects of insulin, whereas hyperinsulinemia under insulin resistance enhances its pro-atherosclerotic effects [35]. Vascular insulin resistance is closely related to endothelial dysfunction, which subsequently leads to atherosclerosis and DN [36]. Insulin promotes vasodilation and inhibits smooth muscle cell proliferation and podocyte apoptosis by stimulating nitric oxide (NO) production [37]. Studies have also shown that insulin intervention aimed at improving blood - glucose control can effectively correct or prevent the hyperfiltration state of diabetic glomeruli. The underlying mechanism is that physiological levels of insulin and blood glucose are essential for maintaining the normal regulatory function of chloride channels. Consequently, in a diabetic environment, dysfunction of this regulatory mechanism is considered a major cause of calcium uptake disorder and subsequent contractile dysfunction in glomerular mesangial cells [38]. The binding of BPA to INS with a binding energy of -6.1 kcal/mol supports the hypothesis that it may interfere with the insulin signaling pathway.

The molecular docking results demonstrated that the binding energy of BPA with six core targets ranged from -6.1 to -8.6 kcal/mol, indicating that the interactions were spontaneous and may have biological relevance. Among these interactions, BPA exhibited the strongest binding affinity with ESR1 (-8.6 kcal/mol), suggesting potential interference with the estrogen signaling pathway *via* competitive binding. Binding to PTGS2 (-7.7 kcal/mol) suggests a possible effect on inflammatory responses. The interactions with MMP2/9 (-7.6/-7.0 kcal/mol) and BCL2 (-7.1 kcal/mol) indicate possible involvement in the regulation of extracellular matrix homeostasis and apoptosis. The binding of BPA to INS (-6.1 kcal/mol) further supports the hypothesis of interference with insulin signaling. These results provide structural evidence for the multi - target mechanism of BPA.

This study achieved several advances in understanding the relationship between environmental pollutants and diabetes. First, a systematic regulatory network including 42 molecular targets was established to describe the association between BPA exposure and DN pathogenesis at the molecular level. Second, molecular docking analysis supported the high-affinity binding of BPA to core targets such as ESR1, offering a new perspective for understanding how environmental toxins interfere with metabolic balance. These findings contribute to the field in three aspects: providing systematic biological insights into BPA-induced nephrotoxicity; proposing molecular hypotheses that can be experimentally tested; and offering an extensible research framework for investigating other environmental factors in kidney diseases.

## CONCLUSION

In this study, a comprehensive computational biology approach was employed to preliminarily establish the association network between BPA and DN. Molecular docking analysis indicated that BPA exhibited predicted binding activity with multiple core targets, with binding free energy values less than -5.0 kcal/mol, suggesting the potential presence of interactions. The enrichment of these targets in key pathological pathways of DN, together with their potential high-affinity interactions, provides a predictive molecular basis indicating that BPA, as an environmental exposure factor, may participate in the occurrence and progression of DN through multi-target and multi-pathway mechanisms.

It should be emphasized that, in the absence of systematic experimental validation, the results of this study are predictive and hypothesis - generating. Through the network toxicology strategy, core targets were identified, and their association with DN was mainly determined through disease database mining, PPI analysis, and bioinformatics enrichment of functional pathways. Although existing data show certain correlations with renal pathological mechanisms, the actual expression patterns and functional significance of these targets in specific renal cell types (*e.g.*, glomerular podocytes and renal tubular epithelial cells) still require verification through further experimental studies.

Overall, this study establishes a preliminary computational framework for exploring environmental pathogenic factors in DN. It also highlights the importance of computational approaches such as network toxicology and molecular docking in generating hypotheses regarding toxicological mechanisms. These predictive findings provide a theoretical basis for subsequent experimental validation, including *in vitro* cell models, gene intervention studies, and animal experiments, and offer molecular insights for assessing the renal health risks associated with BPA.

## Figures and Tables

**Table 1:** Molecular docking analysis of binding affinity between BPA and core DN-related targets.

**Target Name**	**PDB ID**	**Binding Energy (kcal/mol)**	**Key Interacting Residues**
ESR1	1EV3	-8.6	GLU323,PRO324,PRO325,ILE326,LEU327
PTGS2	1XTE	-7.7	PRO84,THR85,PRO86,ASN87,VAL89
MMP2	5TH6	-7.6	ARG19,ILE20,ILE21,TYR23,PRO29
BCL2	4LVT	-7.1	PHE101,TYR105,ASP108,PHE109,GLU111
MMP9	8H78	-7.0	PRO97,ARG98,CYS99,GLY100,LEU188
INS	5F19	-6.1	VAL3,PHE1,VAL2,GLN4,HIS5

## Data Availability

The authors confirm that the data supporting the findings of this research are available within the article.

## LIST OF ABBREVIATIONS

BPA: Bisphenol A
DN: Diabetic Nephropathy
DM: Diabetes Mellitus
T1DM: Type 1 Diabetes Mellitus
T2DM: Type 2 Diabetes Mellitus
CKD: Chronic Kidney Disease
ESRD: End-Stage Renal Disease
ADME: Absorption, Distribution, Metabolism, and Excretion
GO: Gene Ontology
KEGG: Kyoto Encyclopedia of Genes and Genomes
BP: Biological Process
CC: Cellular Component
MF: Molecular Function
PPI: Protein-Protein Interaction
ESR1: Estrogen Receptor 1
AA: African Americans
PTGS 2: Prostaglandin-Endoperoxide Synthase 2
COX 2: Cyclooxygenase 2
MMP: Matrix Metalloproteinase
eGFR: Estimated Glomerular Filtration Rate
HG: High Glucose
NO: Nitric Oxide
